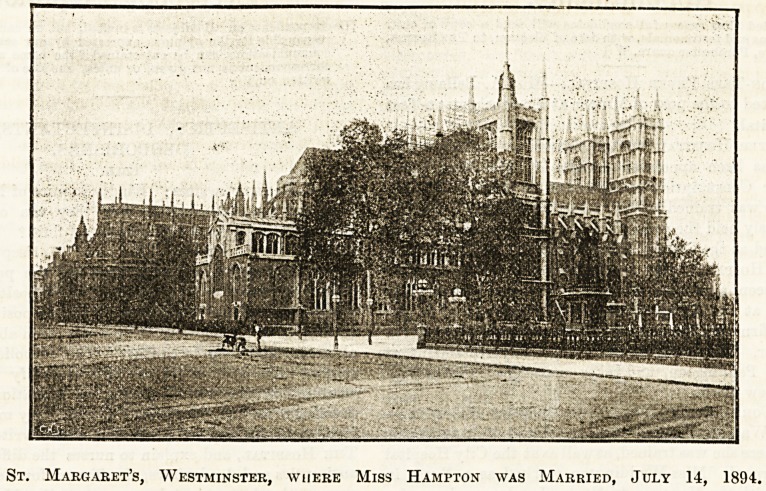# The Hospital Nursing Supplement

**Published:** 1894-08-04

**Authors:** 


"The Hospital" Nursing Mirror.

Being the Extra Nursing Supplement of "The Hospital" Newspaper.

[Contributions for this Supplement should be addressed to the Editor, The Hospital, 428, Strand, London, W.C., and should have the word "Nursing" plainly written in left-hand top corner of the envelope.]

### News from the Nursing World.

### OUR PRINCESS.

Her Royal Highness the Princess of Wales left Charing Cross on Tuesday evening with her daughters, the Princesses Victoria and Maud, for Dover and Brussels. It is anticipated that their absence from England will last about six weeks, as they are to visit the Emperor and Empress of Russia at the Palace of New Peterhoff, near St. Petersburg, and to be present at the marriage of the eldest daughter of their Imperial Majesties.

### PRINCESS BEATRICE AT SOUTHAMPTON.

On Monday last Her Royal Highness Princess Henry of Battenberg paid a visit to Southampton, where she was enthusiastically welcomed on her arrival from Cowes at the Royal pier. The Nurses' Home in connection with the Southampton branch of the Queen's Jubilee Institute was thoroughly inspected by Her Royal Highness, who exhibited much interest in the arrangements made for the comfort of the nurses, their work amongst the sick poor in the town being highly appreciated in the district. It is hoped that the visit of Princess Henry will tend to largely increase the local interest and support given to this excellent association.

### THE LITTLE KING'S HOLIDAYS.

A visit to the seaside is as eagerly anticipated by the young King of Spain as by any little English boy. The grand palace at Madrid, where he generally lives, must be a somewhat gloomy residence, in spite of its fine proportions and the bright modern dwelling. "Miramar," the head-quarters of the Court at San Sebastian, is a far more cheerful abode. The out-door life on the sands with its constantly changing youthful population, has also, without doubt, many attractions for the boy King of Spain.

### MISPLACED DIGNITY.

"The committee thought it would be undignified to pay surprise visits, and did not wish to place themselves in the position of acting as spies," was remarked by the Secretary of the Ladies' Committee of the Hackney Schools during the recent inquiry. The result of the ladies' decision to give notice of their advent was pretty plainly evinced by the Secretary's own opinion, that the poor children had no opportunity of speaking freely without some officer being aware of it. It is hardly likely that any complaints would be made to a party of ladies going formally round, often accompanied by the Clerk of the Guardians, who apparently failed to see that his presence would be detrimental to frank speech on the part of the children. Sick children had no amusement or occupation provided for them, and during at least two years it is said that the children never had one walk outside the school grounds. Yet it is asserted that a rule exists which countenances weekly outings. Surely some of these things might have been remedied if the lady visitors had realised that conscientious visitation of District School children can involve no possible loss of "dignity" on women doing it with the honest intention of improving the lot of the helpless.

### OUR CHRISTMAS COMPETITIONS.

We hope that many of our readers will take away with them a piece of holiday work this year, for we want heaps of things for our Christmas distribution of clothing. The adult patients in hospitals welcome so thankfully the useful gifts sent by our readers, that we annually aspire to see our big parcels multiplied indefinitely. So many patients have to leave the comfortable wards insufficiently clothed in bad times, and the addition of one good warm under-garment often appears to be a necessary accompaniment to the doctor's parting warning as prudence, given to the still delicate breadwinner. The prizes we offer are as follows: For the most serviceable dressing gown made by the competitor, 20s.; for the best flannel shirt, 10s.; for the best flannel petticoat, 7s. 6d.; for the best over petticoat, 7s. 6d.; for the best bed jacket, 7s. 6d.; for the best knitted pair of men's socks, 5s.; for the second best pair, 2s. 6d.

### AN EXCELLENT ASSOCIATION.

The Workhouse Infirmary Nursing Association is badly in need of increased funds, the demand for its nurses far exceeding the supply, and the committee cannot hope to respond adequately to the appeals received unless liberal help is forthcoming. This association is one worthy of all support, the object which it has been advocating steadily for past years being the employment of trained nurses in workhouses. The Hon. Secretary, 6, Adam Street, Strand, willingly gives information.—The Workhouse Infirmary Nursing Association has been applied to by the Guardians of the Newton Abbot Union to recommend a head nurse for their infirmary. They are sending on August 6th Nurse Jane Jeffery, who was trained at the Middlesex Hospital, and was subsequently nurse at the Edinburgh Hospital for Sick Children for two years. She has just completed her midwifery training at Clapham Maternity Hospital, obtaining the L.O.S. diploma.

### PENRITH.

Penrith, with some twelve thousand inhabitants, possesses no hospital of its own. Yet probably people are not exempt from illness or accident there, so they must go to Carlisle, a distance of eighteen miles, if they require hospital treatment. Happily, district nursing is better provided for; the sick are, as far as possible, cared for in their homes.

### LIGHT LITERATURE.

The crowded railway carriages which are steaming out of all the big stations just now, bear a considerable freight of printed matter as well as their living cargoes.

Everybody takes books or papers, or both, to beguile the tediousness of the journey, or "to amuse the children." When all this literature is done with, that is to say, when everything has been read, not necessarily damaged or soiled, it would be gladly received in many a ward or sick room. The out-patients, the frequenters of dispensaries, the hospital patients, and the inmates of workhouses are never likely to have too much light literature at any time. And, by-the-bye, literature for invalids should never be bulky. A heavy volume is as fatiguing as small print to weakly persons, and the small, handy, clear-typed book proves far more acceptable than the costliest well-bound tome.

### TEA OR TABS?

"Well, it depends a little on whether you use tea or tabs," said the lodging-house keeper doubtfully, in reply to a question as to her terms. The applicant looked mystified, and it was some time before she discovered the reason why her ejaculation "Tea!" proved a passport to the desired apartments. But the tabloids to which the landlady objected rejoice the hearts of all nurses, for they consist of concentrated tea, two tabloids of which suffice to brew an excellent large cup, which "standing" cannot render injurious. On a tour where much luggage is inadmissible, one of the neat little tin boxes containing a hundred tabloids will be an acceptable "nurses' companion," the value of which will far exceed the cost thereof. The economy, which consists in making only just the amount of tea required, commends itself more to the nurse than to the seaside landlady, who "don't hold with them tabs."

### NURSES AT GATESHEAD.

A garden party was given last week at Gateshead, in aid of the funds of the Children's Hospital and the Nursing Association. Mr. and Mrs. Walter Wilson opened their beautiful gardens to the visitors, and provided music, refreshments, and entertainments. A sale of work was amongst the attractions, and in spite of bad weather a large number of people assembled in the course of the afternoon. Three district nurses have already accomplished a large amount of work at Gateshead, and there is a general desire to add a fourth to the number. It is hoped that the funds will some day justify the extra expenditure this would entail.

### WITHIN LIMITS.

The nursing of the inmates of Drogheda Union is to be placed in the hands of nuns, and certain important structural alterations are also in contemplation. At a recent Board meeting one of the guardians inquired, "Will they be trained nurses?" to which he received reply, "The nuns would not undertake any duty they would not be competent to perform." It transpired, according to the local press, that they were going to take only the accident cases; neither the midwifery, fever, nor lunatic patients. As it is in contemplation to spend a considerable sum of money on improving the buildings for the sick, the guardians would certainly do well to see that the selected head of the nursing departments is an experienced, fully-trained woman, competent to personally supervise all branches of the infirmary work. If the nuns look after accident cases only, it would be interesting to learn who is to care for the lunatics, the infectious, and the confinement cases, who are also inmates of Drogheda Union.

### AID FOR FRENCH FISHERMEN.

The existence of the hospital ships which during the last few years have added such a valuable department to the Mission to our own Deep Sea Fishermen, appears to be influencing the French to emulate it. The formation of somewhat similar vessels for the convenience of the crews of French fishing boats is strongly recommended in a recent number of the *Progrès Medical*, therefore it is likely that the proposed "boat hospitals" of our neighbours will, in the future, equal our own hospital ships in value and efficiency.

### NOTHING TO DO.

"The days are so long, sir, with nothing to do," replied the infirmary patient, "otherwise I find myself very comfortable." "But surely you can read, and pass the time that way?" "Yes, I can read, and once a week I get a penny paper sent me; but try all I can, sir, it won't last me the week out." "I should think not," responds the visitor, thinking of his own daily and weekly papers, and the pile of monthly magazines at home, "but you should set to work on a cheerful book, which would give you something to think about. By the way, where's the ward library? I know the committee sanctioned the purchase of bookshelves for the ward a year or two ago." The patient said nothing, but pointed to the far end of the ward. The visitor strolled towards the spot indicated, and after a few moments he retired discomfited. The ward library consisted of exactly three volumes, one being Colenso's Arithmetic, another Somebody's "Evidences," and an odd number of a serial. But the visitor returned later on laden with periodicals and cheerful-looking healthy stories, which caused other patients to bless the day when one of the "old chronics" had spoken his mind. A box at the nearest station was shortly provided, and many newspapers were passed on to the ward. "I believe," said a nurse blessed with a sense of humour, "that dread of our ordinary workhouse literature, especially the tracts, adds greatly to the paupers' fear of going into 'the house.'"

### SHORT ITEMS.

Miss Marion Duncan has been appointed inspector of workrooms and laundries at Kensington.—Mrs. Bursnall has been made nurse at the Billesdon Workhouse.—The Local Government Board of Ireland has requested the Guardians of Boyle to find out whether Mrs. Kelly is considered by the medical officer competent to hold the position of infirmary nurse, as "it does not appear that Mrs. Kelly has any qualifications for the position in question beyond a certificate in midwifery."—A garden party was given at Rushenden Hall by the kind permission of Mrs. Sartoris, various entertainments being provided. The local nursing association will benefit to the extent of £12 by this fête which was organised for it.—Miss Marion Gilchrist and Miss Alice Cummings, the first lady graduates of Glasgow University, have passed in all subjects. They were students of Queen Margaret College, Glasgow, before taking up medicine. Miss Gilchrist is an LL.A. of St. Andrew's and graduated "with high commendation."—In Holland there are at present nineteen female medical students.—*Nursing Notes*, for August, contains much interesting matter, including a long article by the Rev. Arthur Peile, president of Queen Victoria Jubilee Institute for Nurses. Full details of the new additions to the Trained Nurses' Club are also given in this number.

### On General Nursing.

By Rowland Humphreys, M.R.C.S., L.R.C.P.Lond.

XXIII.—TYPHOID FEVER—(*continued*).

In fevers, the acid of the gastric juice is deficient in amount or altogether wanting, and its place has to be supplied by the same acid given by the mouth. Pepsin, as a rule, is present in plenty. The pancreatic juice, it seems probable, is deficient in quality, and its powers may be aided by the administration of the liquid obtained, under the name of liq. pancreaticus, from the sweetbread of lower animals.

The food, whether animal or starchy, may also be artificially digested by one of the many methods in use. It should have been said that under the heading of starchy food are included such things as rice, sago, cornflower, bread—all of these can be rendered liquid at the temperature of the stomach by the aid of the pancreatic juice. It is probable that starchy food tends to cause flatulence more than animal food, but the latter, if not properly digested, is more likely to form deleterious compounds. The starchy food, also, has less power to make up for the waste of the body, though it has great heating powers. Starchy food leaves more indigestible residue than meat, and animal food is much more easily absorbed than vegetable food. On the other hand, bacteria grow much more readily in albuminous food than in starchy. It appears (Bunge) that a considerable quantity of the albuminous nutritive material in milk is unabsorbed in passing through the intestines, and it is a matter of daily observation in cases of typhoid that lumps of curds are constantly present in the fæces in spite even of partial predigestion. The artificial preparations of food to be bought ready prepared are not to be taken as of the value indicated in the directions accompanying them. One well-known preparation is simply gelatine, extracted no doubt from meat, but yet only gelatine, and gelatine has but a very small effect on the body; it is said to prevent, to some extent, the waste of the tissues, but cannot be used by the body to renew those tissues. The distinguishing test for gelatine is that when heated it melts, turning solid when cool, whereas albumen turns solid when boiled, remaining so when it cools. Any nutritive preparation, therefore, which does not to some extent turn solid when it is boiled, after shaking it up, if necessary, with a little water, is of little real use to the body. The value of the preparation depends on the proportion of albumen present, and this may be roughly estimated in the same way as the albumen present in urine is estimated, that is by boiling, allowing it to stand for twelve hours, and noticing the proportion the height of the precipitated albumen bears to the column of fluid, allowing for the degree of dilution previously effected.

The choice of food for a typhoid case is, then, based on several things: First, the digestibility of the material; secondly, its nutrient properties; thirdly, its small bulk; fourthly, its heating power; and, lastly, its stimulating action.

Milk appears to contain about 3½ parts per hundred of albuminous material, and rather more of fat, the latter being the best heating material we have.

As compared with lean beef—that is, with little or no fat as it would be if made into real beef extract—the beef contains about twenty-one parts per hundred albuminous material and practically no fat. Only about one-third of that not absorbed by the intestine in the case of milk is here wasted. Thus it would appear that the most concentrated form of food would be a cream of beef or of similar meat food. The cream would furnish the fat, the meat the albumen. About three oz. of albumen are consumed in the body in a state of health in the 24 hours, and this is not increased by muscular work so long as carbo-hydrates (starchy foods) are given in plenty.

The fever is caused by the poison of the disease attacking the heat-regulating mechanism in the brain, and its disturbance gives rise to an increased waste of the bodily elements, the muscles and fat wasting greatly. We naturally try to make up for this waste by the administration of food which is capable of being transformed into the required tissues. The difficulty then arises that the power of digestion as well as the processes of absorption are not working properly in consequence of the febrile state and of the poison circulating in the blood; so that the food tends to accumulate in the stomach and intestines, and its presence and decomposition there will irritate these viscera, and not only cause the signs of gastric and intestinal catarrh (vomiting, flatulent distension, diarrhœa, &c.), but, digestion proceeding further than it should or decomposition taking place, the products of the same are absorbed and give rise to irritation at other points, causing palpitation, sleeplessness, irregular respiration, &c.

In patients who die after a febrile complaint we find that the tissues have not only greatly wasted, but have undergone fatty degeneration. The cause of the fatty degeneration is the want of nourishment, together with the want of oxygen, the life-giving gas inhaled with each inspiration. No doubt the rapid degeneration of the muscles into fat is closely connected with the fever, for the fat gives rise to an amount of heat far in excess of that produced by any other constituent of the body, or by albumen, or by starchy food. An increased bodily temperature necessarily means the production and consumption of a large amount of heat, and it seems reasonable to suppose that the fatty degeneration of the muscles is a natural consequence of the increased demand for heat, and, therefore, fat or predigested starchy food should form a considerable part of the diet of a typhoid fever case. In fever, the regulating mechanism does not act properly, or the temperature would naturally be reduced by free action of the skin. This is imitated by the sponging process now in general use.

As there are only 3½ parts per 100 of albumen in milk, it follows that a healthy person on a purely milk diet requires about four and a-half pints in the twenty-four hours. In a febrile disease this has, of course, to be greatly increased, as bodily waste is greater than in the normal state. This additional amount is made up by the administration of eggs, meat juices, gruel, and such like, in the form of concentrated nourishment. The milk, however, is useful in another way. In fever, the poisons circulating in the blood require removal as quickly as possible. Of course, the bacteria in the tissues and in the intestines are constantly at work making fresh poison, but if the kidneys, skin, and bowels are attended to and kept working, then the poison is rapidly eliminated by them. The water in the milk is of great service in this direction, as it helps to wash the blood, to flush out the kidneys, and to supply the perspiration and fluid contents of the intestines with the necessary amount of water, Milk diet, unfortunately, cannot be taken by all sick people, though many persons, who are quite unable to digest it in health, show no signs of its disagreeing with them when ill. If it disagrees, sometimes removal of the cream (the fat) is necessary; in other cases the cream only can be taken. In other instances, if predigested (as by liquor pancreaticus or Fairchild's digesting powders), or if mixed with barley or plain water, the milk agrees well. Dilution or thickening is always necessary, unless the milk be predigested.

### The Midwives and Trained Nurses' Club.

Very quietly and steadily has the Trained Nurses' Club, at 12, Buckingham Street, grown and flourished. The increased number of members has for some time past made the present accommodation inadequate to the demands on it. Classes, lectures, and meetings have all had to be considered as well as the social aspects of this essentially friendly little club. The committee must therefore be congratulated on their recently acquired additional premises. The new rooms, situated on the same floor, are adjacent to the present ones, and in every respect seem well suited to the requirements of a Nurses' Club.

Buckingham Street, Strand, is such a particularly convenient locality that it is evident that the club will become more popular and useful every year of its existence. Trained nurses and midwives desirous of joining should write for particulars to the hon. secretary at the club, enclosing a stamped and addressed envelope. One of the many useful works of this institute is the maintenance of a registry of masseuses, the names entered thereon being exclusively those of persons whose characters and training are well known to the committee.

### Amongst the Children.

### ISOLATED OR CAGED?

#### Outside.

"Something wrong" with the water supply in the road meant "something right" for the children, who promptly possessed themselves of the immediate advantage of a broad stream which sparkled in the sun. The gutter was flooded, and the news thereof spread to some twenty children, who splashed about in it, free as the birds of the air. None of their clothes were likely to take harm, and the little bare feet pattered in and out rejoicingly on a hot July afternoon.

The children were doubtless "waifs and strays," but as a celebrated preacher said the other day, "it is not well to use those words, nor the popular title 'street arabs.' These are children. Our children," he remarked, "and, therefore, our responsibilities."

#### Inside.

No one has realised this truth more fully, perhaps, than the Sisters at Kilburn, who devote themselves to the service of the orphans, and who are apparently devoted to their self-appointed duties. They house and feed and teach the children in the large and beautiful building devoted to the purpose. But why put the babes into cages? In the event of fire it is impossible to say the extent of the catastrophe which must ensue. The large dormitories are furnished with "cubicles," and instead of these being mere partial partitions to secure a measure of privacy, they consist of four walls of open ironwork encompassing each little bed. There is also a lid or roof of the same construction, which completes the likeness to an aviary or pen, and iron spikes do away with any temptation to unauthorised climbing or scrambling.

The Orphans' Infirmary is provided with similar cages, which are indeed strange objects to find in a sick ward. There is a small lattice on a level with the pillow which can be opened from the outside, and through it nourishment might possibly be administered, but the arrangement is peculiarly suggestive of the trap in the cell door of a prisoner. The object of enclosing any sick person within four iron-work walls is difficult of comprehension. It is impossible that such surroundings should fail to depress nervous or sensitive children and must hinder the work of nursing. Surely, if a little patient is a case for "the infirmary," she needs surrounding with watchful human care and skilled nursing, not to be penned within iron bars. The cage doors in dormitories and infirmary may or may not be kept fastened, but the presence of a padlock suggested the latter was the custom. The kindly and courteous Sisters will do well to promptly consider what terrible death-traps their pretty "cubicles" would become in the event of a fire or other sudden calamity overtaking such a colony of women and children. As regards the moral effect of confining children like wild creatures, much might be said. But that is too large a subject to be entered upon in this place. IQ.

### Notes from Germany.

The mission of the Berlin "Evangelical Local Nursing Association" is in the first place to provide deaconesses for district nursing. At present there are seventy deaconesses engaged in the work, who are, when occasion requires it, assisted by deaconesses belonging to the order of S. Johannes. Eleven branches are worked by from five to eight sisters, who live together in a home provided and furnished for their benefit; they are supported by the ladies of the "Women's Help Association," who keep them supplied with all nursing requisites. During the past year the deaconesses have spent 13,623 days and 3,648 nights in the pursuit of their arduous duties; their patients chiefly consist of women, as the Bielefeld Association of Deacons give their services and devote their time to the nursing of their own sex. The health of the sisters, owing to the many calls made upon their time and strength, is unfortunately not so good as it should be. On an average each sister is engaged in nursing 316 days out of the 365.

Doubtless many nurses in England work equally hard, but the living, both in hospitals and homes in England is, as a rule, infinitely superior to that provided for German sisters and nurses.

According to a recent order of the Upper Austrian Government, certain sanitary precautions are to be brought into effect in Altmünster, Attersee, Ebeusee, Goisern, Hallstadt, Tschl, Mondsee, Schönflink, Seewalchen, Traunkirchen, Unterach, and St. Wolfgang, which are usually crowded at this season of the year by invalids and visitors from all parts of Europe. There are to be isolated houses set apart for the reception of all persons suffering from any contagious diseases, and especially for scarlet fever and small-pox cases. Doctors are in every case and at all times to give immediate notice of the outbreak of an infectious disease to the sanitary authorities. The patients during convalescence are to be placed in strict quarantine, their effects to be thoroughly disinfected before removal; the rooms, after occupation, are also to be thoroughly disinfected and cleansed. A number of officials have already been appointed to superintend the drainage, hygienic arrangements, position, height, and building materials for the new dwellings, to take all precautions against the spread of sickness in any form, and especially of tuberculous disease, and to guard against persons suffering from skin diseases using the ordinary baths, to see that all rooms occupied by the sick are provided with plain wooden furniture and the walls and floors varnished. By request of Dr. Martin Mendelsohn, the editor of the *Zeitschrift für Kraukenpflege*, Dr. Ludwig Jankan, of Munich, has contributed a long and interesting article on "Tobacco Smoking for the Sick and Convalescent." He has made a special study of this subject. Not long ago Dr. Jankan published a treatise entitled "Tobacco, and its Effect on the Human System," and in the above-mentioned article he speaks of the different effect it has upon persons suffering from various diseases, quoting Kubner, who, as is well known, was particularly partial to a cigar.

St. Margaret's, Westminster, where Miss Hampton was Married, July 14, 1894.

### Notes from Victoria.

(By an Australian Correspondent.)

With the approach of winter—which in Southern Australia means the months of July and August—the cry of distress is heard more bitterly, and succour is forthcoming from the charity of the well-to-do, and also in the form of balls in aid of the funds of hospitals and societies. The ball in aid of the District Nursing Society on May 30th realised over £200; that on June 27th for the Austin Hospital for Incurables probably even more. On July 25th the St. Vincent's Hospital—the latest opened in Melbourne—will have a charity ball given in behalf of its funds. A circular, emanating from the Secretary of the Mooroopna Hospital, has been sent to all the hospitals and other charitable institutions, suggesting a conference of representatives of charities throughout the colony to revise the Charities Bill, which is expected to come before Parliament this session. The suggestion has been accepted in most cases, but the date of the conference left open until the Bill is printed and ready for distribution.

The St. John Ambulance Association has now been established in Victoria for eleven years, and seems to have taken a firm hold, especially amongst women, who figured largely as prize-takers at the annual distribution of awards in May, when the medals and certificates were presented by His Excellency Lord Hopetoun. Six hundred and forty-five railway employés and 184 of our police are certificated members of the Association, which, during eleven years, has instructed 7,611 persons in the principles and practice of giving first aid to the injured.

Dr. William Dick, who has for many years been inspector of lunatic asylums for the Victorian Government, in accordance with the age regulations of the Public Service, retired at the end of June, and will be succeeded by Dr. James Victor M'Creery, who for the past twelve years has been the superintendent of the Kew Asylum. Dr. McCreery will still remain superintendent of the Kew Asylum, so that he will have double work to perform, but, in accordance with the present retrenchment policy of the colony, the salary of Dr. Dick, less his pension, will be saved. The arrangement is not regarded favourably by those who understand asylum work even in its most elementary form.

With the cold weather typhoid and diphtheria steadily decrease, but are by no means "counted out." The latest returns are 106 cases of typhoid for a fortnight, with 22 deaths, and 48 cases of diphtheria, with 13 deaths in the same time. Dr. Duncan Turner informs me that the year 1893, the summer of which was cool, was the mildest typhoid year during the last twenty years. Thus comparisons of this year with last are misleading.

Perhaps the increase in typhoid cases during the past summer, which was normal as regards temperature, may be not indirectly due to the enforced parsimony of all the municipalities in the matter of cleaning, but then our population is much less than during last year. Typhoid has appeared in a severe form at the goldfields of Western Australia.

The system of voting for medical officers at the Melbourne Hospital continues to engage the attention of the committee, but proposals seem only made to be negatived. At their meeting on June 19th two proposals were withdrawn and one postponed, and the present defective system is likely to remain in force for a considerable time longer.

The introduction of the plague in the north of Australia by a vessel from China has again brought before public notice the necessity for more quarantine stations.

### Minor Appointments.

Stockton-on-Tees Fever Hospital.—Miss Margaret Martin has been made Charge Nurse at the Stockton-on-Tees Fever Hospital.

South Leith Poor House.—Miss Stevenson, who was trained at the City Hospital, Aberdeen, and Miss Stark, trained at the Edinburgh Royal Hospital for Children, have been made sick nurses at South Leith Poor House. They take with them many good wishes from friends and fellow-workers.

St. Luke's Hospital, Halifax, Yorks.—Miss K. Bruce Biggins has been appointed Charge Nurse at St. Luke's Hospital. She was trained at Stockport Infirmary, and worked as a district nurse at Ashton-under-Lyne, and afterwards did private nursing. Miss Biggins holds the diploma of the L.O.S., and her testimonials are excellent. We sincerely wish her success in her new work.

### Appointments.

[It is requested that successful candidates will send a copy of their applications and testimonials, with date of election, to The Editor, The Lodge, Porchester quare, W ]

Sockton-on-Tees Fever Hospital.—Miss A. Bellamy has been appointed to the post of Matron at the Stockton-on-Tees Fever Hospital.

Manchester Hospital for Consumption.—Miss Eileen Moriaty has been appointed matron of the Manchester Hospital for Consumption and Diseases of the Throat and Chest. She was trained at the Adelaide Hospital, Dublin, and previously held the post of Matron at the Rous Memorial Hospital, and at the Belfast Hospital for Sick Children.

Belfast Hospital for Sick Children.—Miss L. Colborne has been appointed Matron of this institution. She was trained at the Glasgow Children's Hospital and at the Radcliffe Infirmary, Oxford, where she afterwards held the post of Sister. Miss Colborne was Home Superintendent at St. Mary's, Paddington, and takes many good wishes with her to her new work.

Bootle Corporation Infectious Diseases Hospital.—Miss Lillie Waddington has been appointed Matron at this hospital, where she was trained, as well as at the City Hospital South, Liverpool. Miss Waddington worked as a Nurse in connection with the Hanover Institute, London, and was for three years Charge Nurse at the City Hospital South, Liverpool. We wish her every success.

Croydon Borough Hospital.—Miss Alice M. M. Blumenthal has been appointed Matron of this hospital. She was trained at Highgate Infirmary and gained a diploma for midwifery at the Glasgow Maternity Hospital, and as Lady Superintendent of the Barnhill Hospital, Glasgow for four years, and as Matron of the Fulham Infirmary for three years. We sincerely congratulate Miss Blumenthal on an appointment which her extensive experience specially fits her to fill.

Poplar and Stepney Sick Asylum.—Miss Sarah Ann Hannaford, whose appointment as Matron of this asylum was announced last week, held the post of Sister at the Western Infirmary, Glasgow, on the completion of her three years' training in that institution. Miss Hannaford was afterwards made Superintendent of the Nurses' Home and Matron's Assistant. For more than two years she held the responsible position of Matron at the Chorlton Union Hospital, containing 750 beds, where she displayed exceptional administrative ability. Miss Hannaford's testimonials are excellent, and we congratulate the institution which has recently secured her services.

### The Red Cross.

A trained nurse was inquiring the other day under what conditions she would be eligible for adding some kind of badge to her costume, her special ambition being to possess an armlet with a red cross upon it. Any scruples as to the propriety of wearing it were dispersed by a colonial nurse, who explained that she and her friends wore similar decorations simply "because it looks so nice and pretty," and they considered no other justification than this was needed for adopting this decoration. Such a view of the matter naturally, however, fails to find favour in the trained nurse's eyes.

### Liskeard.

The Liskeard Guardians have recently realised that the charge of 54 sick persons by day and by night is too heavy a responsibility for one woman to bear alone. They have therefore consented to give their nurse an assistant, and they offer a salary of ten pounds per annum to a competent person.

### Everybody's Opinion.

[Correspondence on all subjects is invited, but we cannot in any way be responsible for the opinions expressed by our correspondents. No communications can be entertained if the name and address of the correspondent is not given, or unless one side of the paper only be written on.]

### ANTISEPTICS, DISINFECTANTS, AND DEODORISERS.

Izal.

Ellen Hankey writes: Kindly inquire of Newton, Chambers, and Co., Thorncliffe, how they can call "Izal" an antiseptic if it is a non-poisonous fluid? We should all understand the meaning of the word "antiseptic." It means, as far as I know, anything that has the power of killing germs—in other words, against decomposition. It is, of course, the germs which set up decomposition, therefore nothing is an antiseptic that does not mean absolute death to all germs that come in contact. The carbolic spray, for instance. The idea of its use was to purify the atmosphere surrounding the wound during an operation, and at each dressing after the operation. I wish very much that some member of the medical profession would write to this paper The Hospital, and explain to nurses the difference between antiseptics and deodorisers, the former for destroying living germs, the latter to take away smell. Many nurses are ignorant upon this point, and the spread of infection consequently increased by their mistaken idea that Condy's fluid is an antiseptic and a disinfectant, when it is only a deodoriser. They wash out a room containing scarlet fever patients with a weak solution of Condy's fluid, believing it to be as good as carbolic acid and less destructive to their hands. These are questions I would have put to nurses at their examinations. Much better for the public in general if nurses studied the smaller but most useful items which would prove beneficial to themselves as well as to the public, instead of trying to understand Dr. Quain's book on medicine. An antiseptic is a disinfectant, but a deodoriser is not a disinfectant. Kindly enlighten us more fully on this subject. I am sure your reply will be appreciated by many nurses, and, if possible, the reply given in next week's paper.

*** It will be no easy matter to clear up in a satisfactory manner the doubts which exist in the mind of our correspondent with regard to the differences between antiseptics and deodorisers and their respective values—since, according to the loose nomenclature of the present day, the terms disinfectant, antiseptic, deodoriser, and germicide are regarded as interchangeable or convertible expressions. It may, perhaps, assist in the elucidation if we commence by giving a short definition, in the strict scientific sense, of the various terms to which we have above referred. A disinfectant is an agent which destroys the contagium or infection; it does not in the least affect the question to what manner this contagium is so disposed. Thus, for instance, perchloride of mercury is a powerful disinfectant if it comes into actual contact with the virus or poison; thus, if we wash our hands in a solution of perchloride, after touching a fever patient, our hands are disinfected. Or, if we admit free sunlight and fresh air into the room of a similar fever patient the room is usually freed from the contagious or infectious media, and in this sense sunlight and air must be regarded as disinfecting agents. Antiseptics are agents which arrest decomposition, and not, as our correspondent suggests, substances which have the power of killing germs. Decomposition is dependent on the growth of germs; if, therefore, we can inhibit the growth, it stands to reason we stop the decomposition. It is not necessary to kill, although perhaps better to do so, but sufficient to prevent growth, if we want to stay decomposition. And thus it will be understood that any agent which kills is an antiseptic, but the converse does not hold true. To mention a few instances. The germ of decomposition will not grow at very low temperatures. Thus ice is an excellent antiseptic in the case of the great majority of germs. When the ice, however, has turned into water, and a favourable temperature has been reached, decomposition commences. The fact is well known to the purveyor of fish, meat, and fruit, and doctors and nurses are familiar with the arrest of certain infectious diseases by long spells of hard frost. Deodorisers or deodorants are more or less fragrant substances which may mask the odours of decomposition, but need not necessarily destroy the odorous gas or other substances which are the result of the decomposition. Nor need it stop the growth of or kill the germs which may be the origin of the decomposition. Thus tostics or pastilles mask bad odour; they have no other effect. On the other hand, sanitas not only masks the smell, it also destroys it by oxidation, and further acts as an antiseptic by preventing the growth of such germ. A germicide, as its name implies, kills the fully-developed organism or their spores. Perchloride of mercury is a powerful example of this class. Carbolic acid is a less powerful germicide, and permanganate of potash still less powerful. Many substances act in the capacities of all the above mentioned classes. Thus sanitas is a powerful deodoriser, a good disinfectant when properly employed, a fair antiseptic, and a moderate germicide. Now with regard to the question of Izal being an antiseptic, and yet not poisonous, we would remind our correspondent that, what is one man's food is another's poison. What is death to most germs, namely, sunlight, is life to animals and plants of higher development. Everything, perhaps, may be regarded as poisonous if taken in sufficiently large quantities, but what we mean by calling "Izal" non-poisonous, is that as far as human beings are concerned moderate quantities taken internally would not destroy life. And by calling it an antiseptic we imply that in its presence the growth of germs is arrested. We may also add that "Izal" has the further properties of being, to some extent, a germicide and deodoriser. Where other things being equal it is possible to use a germicide in preference to other forms of antiseptic it is very desirable to do so, as by such means we reach the very root of the evil and destroy the foundation of septic or infectious processes.

### MASSAGE.

"E. B." writes: I see in The Hospital of last week, under the heading "Massage," a remark with which I must beg leave to differ, viz., "At present it seems doubtful whether there is an adequate supply of masseurs to meet the demand." I think if reference is made to the consulting physicians, who are the chief employers of massage operators, and to the training hospitals, it will be found that there is an overwhelming supply of very skilful and thoroughly competent masseurs. The disadvantage under which they labour is the licence given by certain departments of the press to more or less trained advertisers who attract not only the viciously-inclined, but also very many who require treatment for various ailments. These accept the women operators as a part of the massage treatment. Such treatment should always be applied, and the certificate of the qualified masseurs and masseuses authorise them only to "apply these methods under medical supervision."

### THE MATRONS' COUNCIL.

Miss Alureda Benger writes: I was present at the Matrons' Council, and though there were differences of opinion and various discussions, yet nothing was done out of order. The matron who writes that "Mrs. Fenwick or Miss Stewart either sat upon or ruled out of order anyone who raised obstacles," has, I think, made a mistake. Miss Stewart endeavoured to be thoroughly impartial, all had a fair hearing. I look upon the Matrons' Council simply as an effort to promote unity amongst us. If we can agree to differ and work with the majority in the main rules of the council, sinking our personal failings for the sake of our work, the aim of the Matrons' Council will be attained. The tendency of a matron's life is to make her become narrow and exclusive; by occasionally rubbing up against each other this will be prevented. We shall all learn something and benefit by our intercourse, we shall widen our sympathies towards one another in our professional work. May I add that the sisters of St. Bartholomew's did not vote when the discussion arose as to whether they or any other sisters should be allowed to join the Matrons' Council as associates; they were in their indoor uniform and easily recognised.

"A Provincial Matron" writes: Bye-law No. 3 is to be brought up at the first meeting of the Matrons' Council in October, and re discussed. The rule has been viewed with so much disfavour, that it has been thought advisable to re-open the discussion, and the matrons who have declined to become members, have been asked to postpone their decision.

### THE SICK POOR.

"Helping" writes: Staying for some weeks in a country village, I have been much struck by the suffering of cottagers during illness—no doctor within miles, and no nurse to be had! I therefore venture to ask the favour of your inserting these remarks in your valuable journal. So many women say they have nothing to do, and get so small an income they can perhaps rent only a single room. Would not some of these good women be willing to help their suffering brothers and sisters? I am sure, if they were now in my place and witnessed the sufferings of a woman with peritonitis, a man with hernia, and no help except from rough, if willing, hands absolutely ignorant of nursing, they would long to do something. "A cup of tea" is the only remedy which suggests itself here for every ailment! There is one lady, far from rich, but she would be glad to take in another lady for a very small sum per annum; and I am sure there are many others who would do the same if they could find sensible women with hospital training to live with them (untrained would be useless), for willing, capable hands, ready wit, and loving sympathy are required, and would find a large field of usefulness before them. Anyone wishing information on this particular village and the lady mentioned can have it; but in the first place I hope someone with a readier pen than mine will express an opinion. People are apt too to fancy themselves of no use in the world, forgetting that "even a cup of cold water" is not given in vain.

*** We do not think many competent trained nurses will be found amongst the women who are content to live in one room and say "they have nothing to do." The people thus situated are generally those who do not possess either the will or ability to carry on any kind of work systematically and unselfishly. However, there may be notable exceptions, and if so we trust someone will respond to "Helping."

### Novelties for Nurses.

### A NEW FORM OF CORSET.

(Herts and Co.)

We have been sent a specimen of a most admirable substitute for the ordinary corset, which we hasten to recommend to the attention of our readers. This new form of corset is, in reality, corset and slip bodice combined. Women of all classes must be sensible of the disadvantage of wearing as part of their underclothing an article which cannot be consigned to the laundress every week. For this reason coloured corsets are often worn to disguise the soiling which must take place in time, and very rapidly when worn in London. This compromise is not very commendable. If all corsets in the future were made as Messrs. Herts have arranged in their "Platinum" anti-corset there will be nothing left to be desired, and all difficulties will be obviated. The "Platinum" corset-bodices are in effect bodices "boned," so to speak, with platinum supports. These platinum "bones" are all made removable, and slip out out and into the seams with perfect ease, their removal, when the bodice is sent to the laundress, occupying about a minute. The "bones" are neatly covered, are thin, pliable, and durable. We can only wonder that so obviously practical and excellent an arrangement was never thought of earlier. Of course, two corsets will be necessary, but they will last as long as two corsets under ordinary circumstances; and, as the prices are most moderate, no difficulty should arise in their adoption. The idea is an excellent one, and to be recommended in every way.

### A Holiday in Switzerland.

[By B. E. W.]

A little while ago the Editor of The Hospital invited anyone who enjoyed a pleasant holiday at a moderate cost to give an account of it for the benefit of others who might wish to go and do likewise. As our little trip this spring was a delightful one, nurses may like to have the details of

Twenty-eight Days by Lake Geneva for Eleven Guineas a-head.

We did not go with the intention of doing much sight-seeing, but people can always suit themselves and their pockets about that. There are plenty of charming trips to be made from any of the places. The early time of year we chose for our trip helped to make it cheaper. We started just before Easter, and prices are not generally raised till May.

Our money was laid out as follows: Return ticket to Clarens, from Cook's, for forty-five days, £4 15; *pension*, weekly, 25s. (31 frs., or 4 frs. 50 a day), £5; leaving £1 16s. for pocket money for the month, out of our £11 11s. About 5s. for the journey each way is required for cabs, tips, refreshments, &c. We had second-class accommodation and found it quite comfortable, our only trouble being the difficulty of getting anything to eat without losing the train. Another time we shall take more provisions with us, as one really substantial meal is needed, besides oranges, chocolate, and cake or biscuits; *Café au tait* and nice little loaves being the only provisions obtainable in the train. Perhaps, in a "personally conducted" tour, these little things are looked after, but we thought it was better fun to do it all for ourselves.

The journey takes twenty-four hours, straight through by Newhaven and Dieppe, Rouen, Paris, Dijon, Poutarlier, Lausanne, and then Clarens.

They say it is possible to travel without speaking French, but I think it must be very awkward to require an interpreter on the railway. Perhaps owing to our provincial accent, our cabman landed us at the wrong terminus, for the garçon told him we wished to go to the Gare d'Orleans, while I intended to impress on him to order the driver to take us to the Gare de Paris à Lyons. Happily the two stations are not far apart, and "all's well that ends well."

Readers will think we have skipped the English Channel pretty easily, but that is rarely a pleasant subject; suffice it to say three out of four were prostrate below decks, while the fourth was rendered very conceited by braving the rather rough weather (the decks being thoroughly wet with spray though a bright sunshine kept up the spirits), and none were the worse for the winds and the waves. Certainly fresh air and pluck are the best preventives of sea sickness on such a short voyage—four and a-half hours.

We found Clarens a lovely spot amidst most beautiful surroundings, the air clear and fresh though quite warm, a swift mountain torrent rushing down into the calm blue waters of the lake, pretty villas and fine chateaux all along its shores or perched among the vineyards on the hills which culminate in pine-clad and snowcapped mountains. "Climbing made easy" is the motto in these parts, and no one needs to be a mountaineer to enjoy it; electric tramways pass the door and take passengers to funicular railways that transport them to a great height to revel in grand scenery. The lake steamer carries one across the blue waters to Savoy or up to Geneva. A saunter up the hill-side, picking white violets and wild orchids, to the little village of Chailly is delightful, or higher up Blonay, with its Castle of Chillon. Of course one must visit and go shopping in the lively town of Montreux and the quaint market place of Vevey. We found it best to breakfast early and start out at once, as dinner was at half-past twelve. Then we would amuse ourselves and rest till we felt inclined for afternoon tea, which we provided for ourselves, and then we went out again till half-past six supper. After that we could spend the evening with the other visitors in the salon at halma, chess, needle-work, music, and conversation, or we would write our letters and chat in our own rooms. The drawback at Clarens is that almost all visitors are confirmed invalids, so there are none of those charming excursions such as are generally planned, when Madame packs a cold lunch, and the party sally forth very early for some well-known spot, to come back brown and tired, but not over-tired. The invigorating air lends strength even to indifferent walkers. There is no more healthful way of spending a day than amidst the delicious sights and sounds that abound on all sides in this lovely country, and seem to make life worth living indeed.

After ten days here we sought a more bracing climate in Lausanne, a favourite place when we were at school there a good many years ago. Since then it has grown and improved, but has still the same charm; so bright and cheerful, such an open situation, such steep, old-fashioned streets, such fine wide roads in the new part of the town, such lively young students in their different coloured caps, such a mixture of French, German, English, and Americans, and other nations, and such delicacies in the way of cakes, caramels, and meringues, and such an appetite for eating them, as surely no other town possesses!

"Hot and dusty and too steep" is the verdict of some, but the air gives such vigour that dust and steepness cease to be considered as drawbacks. One day in the country here was spent thus: The hostess (an English lady) and I breakfasted at seven, and started for Croy Romainmotier, a mountain village, about twenty miles out, and tolerably high up. The train was a very slow one, and seemed uncommonly full of small children; quite a crèche in fact. A very agreeable nurse told us they were all going to a convalescent home at Edepines, belonging to some sisters who have a crèche at Nyon. When we got out at Croy, we took the diligence up the hill to the old country house we were going to visit, formerly belonging to an old Swiss family, whose portraits still adorn the walls. Being Catholics, they had also a small chapel adjoining; this has since been used for theatricals and billiards, and is now to be turned into a salle à manger by our practical hostess for her guests. In the afternoon the young people took me for a delightful ramble to the source of La Sarrez, a stream which bubbles out of the woods clear and cool; and then up the hills, whence we had a fine view towards Neuchatel and the Bernese Oberland, with its many white-capped summits. There were more delights than these, but I feel I must draw to a close. We returned in couples, had a capital journey, leaving Lausanne after supper at eight o'clock one evening, breakfast at Paris eight o'clock next morning, and home to supper at eight o'clock in the evening. As we had perfect weather all the time, and spent no more than we meant, we all voted our trip a great success, and have no objection to giving addresses of *pensions* or further particulars if desired by any reader of The Hospital.

### A Case for Assistance.

Contributions of 3s. from Sister Rose, 2s. 6d. from An Old King's Nurse, 1s. 6d. from Miss W., making total up to date £4 0s. 6d., have been received by Miss Bell and Miss Farrow, of "The Liverpool Homes for Aged Mariners, Egremont, Cheshire," for the nurse whose sad case has been stated in our columns. Further donations are urgently needed to enable her to furnish the little shop at Cardiff, where she hopes to earn her own living.

☞ For The Muses' Looking-Glass, Reading to the Sick, Book World for Women and Nurses, &c., see page clxxxix. et seq.

### The Muses' Looking Glass.

### ROUND CHARING CROSS HOSPITAL.

ONE is inclined to inquire, "Can any good thing come out of Bow Street?" or, "What possible interest, except to criminals, can be attached to the office of a police magistrate?"

In spite of its sordid associations, however, if we look into the Bow Street of a hundred and twenty years ago we may catch a glimpse of romance and pathos in the disease-stricken figure of Henry Fielding, sitting in judgment upon the depraved habitués of his Court, but, during his intervals of leisure, passing severer sentence upon himself for his conduct to his first wife, and publicly proclaiming his own condemnation in the pages of "Amelia."

While one would be sorry to recommend Fielding's novels for indiscriminate reading, the most rigid upholders of the censorship owe nevertheless a debt of gratitude to the great prose Homer for his influence on Thackeray; and that he did something for the purification of morals in his own day one gathers from Mallet's lines, supposed to be uttered by Tyburn Tree—

"Fielding, too—a mischief on him!
I wish my sons would meet and stone him!—
Sends his black squadrons up and down,
Who drive my best boys back to town,
They find that trav'ling now abroad
To ease rich rascals on the road
Is grown a calling much unsafe,
That there are surer ways by half . . .
Of earning daily food and fame;
So down, at home, they sit and think
How best to rob with pen and ink!"

His official efforts to put a stop to highway robberies may account for the fact that Fielding's house was pulled down in the Lord George Gordon Riots on June 9th, 1780, as Dr. Johnson, writing an account of this outburst of indignant Protestantism to Mrs. Thrale, informs us, adding, the mob "burnt his goods in the streets."

We can hardly imagine the magistrate was suspected of Popery, but an earlier member of the family who claimed kinship with the Hapsburgs, and was celebrated for his beauty during the reigns of three kings, was reconciled to the Catholic Church during the days of James II. This Beau Fielding was the victim of a practical joke, played upon him by a frolicsome lady in consequence of his frequent attempts to marry rich widows. Mrs. Wadsworth lived near Leicester House, and Fielding, believing her to be worth a fabulous fortune, fetched a Catholic priest from that historic mansion one day to solemnize a marriage between them. When undeceived he turned his wife out of doors, and three weeks afterwards married Charles II.'s old mistress, the Duchess of Cleveland, but after a short experience of her handsome husband Duchess Barbara provided Mrs. Wadsworth the money to prosecute Fielding for bigamy.

In an extant map, which was probably planned about 1592, all the region north of Charing Cross and west of Chancery Lane is represented as truly rural; Covent Garden was the walled orchard of the Abbots of Westminster; and although Abbots were gone by Queen Elizabeth's time, milkmaids with pails on their heads still tripped along the footpath over Lammas Fields, and farm dogs had a delightful run at the maids' heels where now stand the carriage shops of Long Acre.

The Lammas lands belonged partly to the Abbey of Westminster and partly to the Leper Hospital, founded by Matilda, Henry I.'s Saxon wife, and dedicated to St. Giles, the patron of lepers. St. Giles's hospitals will generally be found outside towns, and St. Martin's Lane is marked on this old map as a track running from the Strand past St. Martin's-in-the-Fields to St. Giles's Hospital, and thence to the Marylebone Woods.

Many illustrious people have lived in the neighbourhood of Leicester Square, and Leicester House was frequently a home of sadness. The little Duke of Gloucester and his sister Elizabeth were placed by Parliament under the charge of the Sidneys here during the imprisonment of their father, Charles I.; and their aunt, the ill-fated "Snow Queen," or "Queen of Hearts," as she was called (from whom our new infant prince derives his claim to succeed to the throne of the Stuarts), found a refuge from debt and trouble in this house, lent to her during the reign of Charles II. The poor lady experienced a warmer welcome from her many admirers than from her royal nephew, if one may judge from the story of her arrival in England from Holland.

George II. bought Leicester House and retired here when, as Prince of Wales, he had quarrelled with his father; and the traditions of the House of Hanover requiring a feud between successive Georges and their heirs, here also Prince Fredrich held his opposition Court. Addison's play, "Cato," was acted at Leicester House by Fredrich's children; little Prince George—afterwards King—played the part of Porteus, but the life their mother led may be guessed at from an anecdote about these children which has been preserved.

"Can you tell me, Eddy," the Princess of Wales asked one of her little boys, "what a pronoun is?" "Of course I can," replied the child, "a pronoun is to a noun what a mistress is to a wife—a substitute and a representative."

The "Golden Head" was the sign of Hogarth's house in Leicester Fields, and John Hunter, whose memory St. George's Hospital has been celebrating recently, lived behind the Square. There is a story of Goldsmith, which we will quote at the risk of relating what is well known, because it affords a peep at Leicester Square in the 18th century.

Edmund Burke, accompanied by an Irish officer, was walking through the Square one day, on his way to dine with Sir Joshua Reynolds, when he saw Goldsmith bound for the same hospitable board, but too much taken up with regarding the groups of people staring at some foreign women at an hotel window to notice Burke.

"Look, O'Moore," said the statesman, "there's Goldsmith; by-and-by at Reynolds' you'll see what I'll make of this."

Arrived at Sir Joshua's Burke treated his fellow guest with marked coldness. Poor Noll, who had the most affectionate heart in the world, was hurt, and inquired the reason.

"No wonder, Mr. Goldsmith. What can you expect after the monstrous indiscretion on your part which I witnessed in the Square just now?" remarked Edmund. Goldsmith, astonished, inquired what he meant. "Why, didn't you exclaim, 'What stupid beasts the crowd must be to stare at those painted Jezebels, while a man of your talent passed unnoticed?'" "Surely, my dear friend, I did not say so?" "If you had not, sir, how should I have known it?" "That's true," replied Goldsmith, humbly; "I am sorry; it was very foolish. *I own something of the kind did* pass through my mind, though I didn't think I uttered it!"

At the corner of Bow Street and Russell Street, awe-struck country visitors to London in the early part of last century might catch a sight of the great Dryden passing into Wills' Coffee House. From Wills', too, in later days, the dramatic criticism of the *Tatler* was appropriately dated, being near enough to Drury Lane to enable the writer to pen his remarks upon the tragedy with the glamour of the stage still over his senses.

Addison, in 1712, started Barton's Coffee House in Russell Street, and here, in Johnson's time, letters for the *Guardian* might be dropped into the lion-headed box designed by Hogarth in harmless mimicry of the sterner institution at Venice.

One of Goldsmith's little humorous poems is addressed to "Iris, in Bow Street, Covent Garden." "Cruel Iris" evidently only valued her admirers for what they were worth in presents, and little was to be got out of poor old Noll. "If gems, or gold, impart a joy, I'll give them—when I get them," he writes, and concludes his addresses to the fair one thus:—

"I'll give thee something yet unpaid,
No less sincere than civil;
I'll give thee—ah! too charming maid,
I'll give thee—to the devil!"

### For Reading to the Sick.

### GOD'S COMPASSION.

**Motto.**

Sweet are the uses of Adversity.—*Shakespeare.*

**Verses.**

Only those are crowned and sainted,
Who with grief have been acquainted.—*Longfellow.*

Who is the angel that cometh?
Pain!
Let us arise and go forth to greet him;
Not in vain
Is the summons come to meet him;
He will stay,
And darken our sun;
He will stay
A desolate night, a weary day,
Since in that shadow our work is done,
And in that shadow our crowns are won!
Let us say still while his bitter chalice
Slowly into our hearts is poured—
Blessed be He that cometh
In the name of the Lord.—*A. Procter.*

**Reading.**

I gazed on my moth as it struggled to free itself from its prison, and thought I was wiser than the moth's maker, and resolved to give it a helping hand.

With my scissors I snipped the confining threads to make the exit easier, and lo! immediately and with perfect ease out crawled my moth.

In vain I watched to see that marvellous process of expansion in which those shrivelled wings silently develop.

But I looked in vain. My false tenderness had proved its ruin. It never was anything but a stunted abortion, crawling painfully through its brief life which should have been spent flying through the air on rainbow wings.

The lesson I got that day stood me in good stead. It has helped me to understand what the Germans mean when they speak of the "hardness of God's love."

I have often thought of it when watching, with pitiful eyes, those who were struggling with sorrow, suffering, and distress, and it has seemed to me that I was more merciful than God, and would fain cut short the discipline and give the deliverance.

Short-sighted fool! How know I that one of these pangs or groans could be spared? The far-sighted, perfect Love, that seeks the perfection of its object, does not weakly shrink from present, transient suffering. Our Father's love is too true to be weak.

Because He loves His children He trains them that they may be "partakers of His holiness," "made perfect through suffering," as the Elder Brother was.

The sons of God are trained to obedience, and brought to glory through much tribulation. We are in the hands of a higher Physician than this world knows—One who cannot mistreat our case or prescribe wrongly for us. The great cure to be wrought in us is the cure of self-will that we may learn self-resignation, and all God's various dealings with us have this one end in view.

And what happiness is it to attain to this—to the perfect knowledge and feeling that we are in the hands of a compassionate Father, who cares for our every want, and supplies it too, only in the way His unerring wisdom knows to be best, but which our short-sightedness would rebel against because we cannot see how it should be so. We are in His hands; His will be our will. If He sees fit, welcome sickness, for in Him alone is health. We have the blessed consolation that whom He loveth He chasteneth, and that He chasteneth that we may not be condemned with the world. Believe it, in sickness or weakness, providing we submit to His will, He teaches us more blessed truths than years otherwise would suffice to acquaint us with.

### The Annual Meeting of the Royal British Nurses' Association.

Favoured by brilliant sunshine and a cloudless sky, the Royal British Nurses' Association held its annual meeting at Windsor last Wednesday. An unusually large number of members and their friends were present on the occasion, as, in addition to the well-known attractions of the Royal borough, exceptional privileges in the way of sightseeing had been obtained through the instrumentality of Her Royal Highness Princess Christian, President of the Association. On arriving at Windsor the members proceeded at once to the Guildhall, where the preliminary business was transacted. At twelve o'clock Princess Christian, attended by Baroness von Egloffstein, drove up from Cumberland Lodge, and was received on alighting by Sir W. Savory, Dr. Fairbank, Dr. Bezly Thorne, Mrs. B. Fenwick, Mrs. Spencer, and Miss de Pledge. H.R.H. opened the proceedings by welcoming those present to the home of her childhood, expressing a wish that the day might remain a happy memory to all present. A sumptuous luncheon, afterwards enjoyed by 220 guests in the Albert Institute, was provided by the kindness of the Mayor of Windsor and other influential gentlemen of the neighbourhood. After the toasts, "The Queen," "H.R.H. the President," and others had been proposed and received enthusiastically, the company dispersed, and parties to various places of interest were organised. By the special permission of Her Majesty the beautiful mausoleum at Frogmore was inspected by a limited number, who were conducted round by Princess Christian. The Royal gardens and model dairy were next visited, also St. George's Chapel, the State apartments, and famous library. The wealth of treasure exhibited in the latter embraces contributions from all nations and all ages, and arrests the admiration of even the unlearned. The members partook of tea, by the special invitation of H.R.H., at the Trained Nurses' Institute, Clarence Villas, where a tempting repast was found awaiting them. The interest taken in this home by Princess Christian, its foundress and patron, is well known, and she has rendered it well-nigh perfect of its kind. The bedrooms are tastefully decorated, with polished floors and comfortable furniture. Everything breathes of home and refinement.

Advantage was taken of a beautiful evening to prolong the day's pleasure by excursions on the river and walks in the neighbourhood. Not till it was quite dusk did the members of the party think of retracing their steps homeward, after one of the most successful gatherings ever held under the auspices of the Association.

### Notes and Queries.

The contents of the Editor's Letter-box have now reached such unwieldy proportions that it has become necessary to establish a hard and fast rule regarding Answers to Correspondents. In future, all questions requiring replies will continue to be answered in this column without any fee. If an answer is required by letter, a fee of half-a-crown must be enclosed with the note containing the enquiry. We are always pleased to help our numerous correspondents to the fullest extent, and we can trust them to sympathise in the overwhelming amount of writing which makes the new rules a necessity. Every communication must be accompanied by the writer's name and address, otherwise it will receive no attention.

**Queries.**

(106) *Medicine.*—Kindly tell me whether you consider a Glasgow degree would enable a woman student to secure in the future a good appointment.—*Meath.*

(107) *Dipsomania.*—Wanted, the address of a home for a lady. Must be a well-appointed house.—*Nurse.*

(108) *St. John Ambulance.*—Kindly give me information as to the nursing dress of this Association.—*Mrs. G.*

**Answers.**

(106) *Medicine (Meath).*—Certainly it would, if the qualified woman were in other respects eligible for the post sought. Your query respecting "manners" would hardly have been put had you realised how much each individual woman can do to raise the standard.

(107) *Dipsomania (Nurse).*—Write to Dr. Stewart, Dunmurry, Sneyd Park, Clifton, Bristol.

(108) *St. John Ambulance (Mrs. G.).*—You had better write for all particulars to Lady Secretary, St. John's Gate Nursing Division, Clerkenwell, London.

### The Book World for Women and Nurses.

[We invite Correspondence, Criticism, Enquiries, and Notes on Books likely to interest Women and Nurses. Address, Editor, The Hospital (Nurses' Book World), 428, Strand, W.C.]

Katharine Lauderdale. By F. Marion Crawford' Three vols. (London: Macmillan and Co. 1894.)

We cannot say that this book impresses us as being altogether equal to the author's deservedly great reputation. It gives too much the idea of a story made to order—to fit a frame, as it were—and has certainly suffered in being spun out into three volume length. The story is not complex. The scene, like that of "Marion Darche," is laid in America, and the opening dialogue introduces us to the hero, John Ralston, "dark, good-looking, nervous, excitable, enduring, and decidedly dissipated at the age of five-and-twenty years," A representative of an old Scottish family, long settled in the States, he is justly proud of his race, and a high sense of honour is a special characteristic, but—and the plot of the story centres round this failing—"he was said to drink," and with truth, though his strong constitution is able to throw off its effects, leaving apparently "not a wrack behind." His very efforts to struggle with this besetting weakness, through a series of untoward events, lead him to the forfeiture, for the time of his mother's and his *fiancée's* confidence, they, like all the New York world, believing that a quarrel with his best friend, Hamilton Bright, an accident, and a fight with a professional boxer, which result in his being picked up by the police and taken, unconscious, to his mother's house, are the consequences of a drunken row. Katharine Lauderdale, to whom he is secretly engaged, is charming, and the contrasting characters of herself and her mother, and a somewhat crabbed father, are powerfully drawn. How Katharine persuades her lover, against his better judgment, to a secret marriage, and how through the testimony of a doctor friend he is finally cleared from the imputations which have attached to him, and all comes right in the end, we must not spoil the interest of the story by telling. The relations between Jack Ralston and his mother, and the good fellowship and sympathy which exist between them, are pleasantly brought out, though here we think the author hrdly does justice to the mother in making her refuse to believe her son's word of honour. Mr. Marion Crawford hints that there will be a continuation of the history of Katharine and her husband, which cannot fail to be welcomed by the author's many admirers, the end of the present story leaving the reader still unsatisfied on more points of interest than one. Notably, we should like to hear more of that unpleasantly magnetic person, Walter Crowdie, the artist.

"How to Rear Healthy Children: The Baby" is a good sound leaflet which can be relied on as safe and profitable reading for an inexperienced mother or child's nurse. It is issued by the Ladies' Sanitary Association, 22, Berners Street, at 1s. per packet of 50 copies.

---

### MAGAZINES OF THE MONTH.

Cornhill Magazine.—This month's number contains some good contributions. "Gleams of Memory; with Some Reflections," by James Payn, is continued. Speaking of the relations between authors and critics, he says, "There is a great deal of bad feeling among authors in respect to criticism, which mainly arises, I think, from an exaggerated estimatc of its power for good or evil; whereas it can do little good to a bad book and little harm to a good one." After a detailed discussion of the special complaints of novelists, he comes to the conclusion that the author's] grievance is "not a very grave one." "The Happiest Man in London" is a touching sketch of an East-end old couple. The wife was paralytic and bedridden; the husband, besides his daily work, has to nurse her and do all the cooking and home duties; yet the best wish he can give a rising young doctor about to be married is that his married life may be equally happy! The "Character Note" is a very smart little sketch of a fading London beauty. The grim pathos of the sketch is that every woman, while despising this character as portrayed by the writer, must feel a sympathy prospective or retrospective with her feelings.

Cassell's Family Magazine.—There are several nicely told stories in this number and some good short sketches. Now that there is talk of municipal pawn-shops in London, Mr. Fred Barnard's illustrated account of pawnbroking as it is at present is timely. He calls it "At the Sign of the Golden Pills," for the familiar three balls are simply the family crest of the Italian de Medicis. A story is told of a North-country village, where strikes had caused much depression and there was unusual resort to "my uncle's." The pawnbroker's shop was burnt down on a Saturday, and it was noticed that very few of the labouring men, who were unusually regular attendants at divine service, were in their wonted places, while the lay preacher himself performed his duties in his week-day clothes.

The Humanitarian, as usual, contains a medley of articles by faddists, and brightly-written thoughtful articles. Of the last, one of the most interesting is by Mr. Douglas Sladen on "The Position of Japanese Women." In most cases a Japanese bride is taken to the paternal home of her husband, and is expected to be the docile, good-humoured, and attentive servant not only of her husband but of her father-in-law and mother-in-law. Even the most high-born women have to make and mend the clothes of their husbands; they must rise first of all the household, arouse the servants, tidy the house, take hot water to the rooms of the husband and of the parents-in-law, and prepare breakfast. "A wife must look to her husband as lord, and must serve him with all worship and reverence, not despising or thinking lightly of him. The great lifelong duty of a woman is obedience." Divorce is very common in Japan, and carries with it so little stigma that men and women may be divorced and married several times over. But in accordance with the Japanese theory that heredity goes only with the male line, the children always remain with the father.

The English Illustrated Magazine has a very bright and interesting August number. It contains illustrated interviews with two people so different as Fry, of Wadham, the celebrated young Oxford athlete, and Professor Blackie, of Edinburgh, the *doyen* of Scottish learning and culture. An interesting account is given of life on board a torpedo-catcher. The multiplication of torpedo boats in the navies of Europe has made necessary the existence of a special class of very fast steamships to capture the torpedo boats. Although they have not yet been tried in actual warfare, the experience gained in naval manœuvres shows that they are well adapted for their purpose. But life on board them must be very uncomfortable, for nearly everything has been sacrificed to speed. The quarters of officers and men are almost equally confined, and the boats roll so easily that even in harbour the wash of a passing steamer sends everyone staggering from his place. An account of "Recent Advances in Landscape Photography," with beautiful illustrations, shows how photography is rapidly becoming a branch of art. Those who add amateur photography to the pleasures of a holiday can see here how much better things there are to do than to take casual "snap-shots" at anything coming in their way. There are several good short stories, the best of which is "Her Son's Wife," by Clara Savill Clarke.

## Figures and Tables

**Figure f1:**